# Low-Rank Tensor Fusion for Enhanced Deep Learning-Based Multimodal Brain Age Estimation

**DOI:** 10.3390/brainsci14121252

**Published:** 2024-12-13

**Authors:** Xia Liu, Guowei Zheng, Iman Beheshti, Shanling Ji, Zhinan Gou, Wenkuo Cui

**Affiliations:** 1 School of Management Science and Information Engineering, Hebei University of Economics and Businesses, Shijiazhuang 050061, China; liux@hueb.edu.cn (X.L.); zhinan.gou@hotmail.com (Z.G.); wenkuo@tongji.edu.cn (W.C.); 2 School of Computer Science and Technology, Harbin Institute of Technology, Weihai 264209, China; zhenggw@stu.hit.edu.cn; 3 Department of Human Anatomy and Cell Science, University of Manitoba, Winnipeg, MB R3T 2N2, Canada; 4 Institute of Mental Health, Jining Medical University, Jining 272111, China; jishanling@mail.jnmc.edu.cn

**Keywords:** brain age, spatial–temporal, multimodal, low-rank tensor fusion, machine learning, deep learning

## Abstract

**Background/Objectives:** A multimodal brain age estimation model could provide enhanced insights into brain aging. However, effectively integrating multimodal neuroimaging data to enhance the accuracy of brain age estimation remains a challenging task. **Methods:** In this study, we developed an innovative data fusion technique employing a low-rank tensor fusion algorithm, tailored specifically for deep learning-based frameworks aimed at brain age estimation. Specifically, we utilized structural magnetic resonance imaging (sMRI), diffusion tensor imaging (DTI), and magnetoencephalography (MEG) to extract spatial–temporal brain features with different properties. These features were fused using the low-rank tensor algorithm and employed as predictors for estimating brain age. **Results:** Our prediction model achieved a desirable prediction accuracy on the independent test samples, demonstrating its robust performance. **Conclusions:** The results of our study suggest that the low-rank tensor fusion algorithm has the potential to effectively integrate multimodal data into deep learning frameworks for estimating brain age.

## 1. Introduction

Brain aging is the gradual decline of mental function. The “brain age” biomarker measures the aging status of the brain [1]. Advanced machine and deep learning techniques, combined with brain imaging scans, are used to derive brain age [2,3,4,5,6,7]. Studying brain aging can help identify markers that indicate its progression.

Functional magnetic resonance imaging (fMRI), structural magnetic resonance imaging (sMRI), diffusion tensor imaging (DTI), and magnetoencephalography (MEG) have been instrumental in detecting age-related changes in the brain [8,9,10]. Among these modalities, sMRI is commonly used to estimate brain age due to its high-resolution images that enable tracking of structural brain changes [10,11,12]. Moreover, sMRI data are more widely available than other modalities, enhancing the reproducibility of brain age research [13]. For instance, Cao et al. applied the least absolute shrinkage and selection operator (LASSO) algorithm to longitudinal sMRI data from 303 healthy controls (HCs) for predicting individual brain maturity [14]. Beheshti et al. introduced a unique 3D patch-based grading procedure for estimating cortical aging using sMRI data [15,16]. Franke et al. presented a framework for efficiently estimating the brain age of 650 HCs from their sMRI scans using a kernel method for regression [17]. Valizadeh et al. employed sMRI data from 3144 HCs to extract various anatomical features and using them to predict age through different statistical techniques [18]. Cole et al. used convolutional neural networks to estimate brain age using raw sMRI data from 2001 HCs [19]. Lancaster et al. trained a Bayesian optimization framework with data from 2003 HCs to predict age [20]. Liu et al. constructed a multi-feature-based network (MFN) to estimate the brain age of 2501 HCs by describing structural similarities between traditional cortical morphological features [21]. Liem et al. assessed functional connectomes and mean time series from both cortical and subcortical regions, using support vector regression and regression based on random forest methodology to predict brain age [22]. Martina J. Lund employed resting-state fMRI data from 1126 HCs to estimate functional connectivity between brain networks, using these as features to predict brain age [23].

In addition to the aforementioned techniques, DTI enables the identification of diffusion and topological patterns across diverse brain regions, thereby aiding in the prediction of aging [24]. Benson Mwangi et al. applied a multivariate technique, relevance vector regression, to predict age using features extracted from diffusion tensor imaging [25].

Previous studies have primarily estimated brain age using single-modal neuroimaging data. Research has demonstrated that data fusion among data from various imaging methods could provide a more robust machine learning model and also provide a more comprehensive understanding of brain function, structure, and connectivity [26,27]. In the area of brain age estimation, recent research studies have also focused on integrating features from multiple modalities, demonstrating improved accuracy in brain age prediction [28,29,30,31]. For instance, D.A. Engemann et al. combined MRI, fMRI, and MEG features to estimate brain age [32].

It is well known that the T1 signal intensity of brain structures varies with age due to changes in brain tissue composition [33,34]. DTI helps to detect changes in diffusion and topological patterns in the brain associated with aging [24]. Thus, studies using unimodal features often fail to simultaneously account for age-related functional and structural changes in spatial and temporal domains, which could potentially improve prediction performance. Therefore, the combination of sMRI and diffusion images with functional metrics (such as EEG/MEG or fMRI) holds promise for enhancing brain age prediction. However, a key challenge in developing multimodal brain age estimation frameworks is the effective integration of data from diverse sources. This integration is crucial for improving prediction performance and providing a comprehensive view of structural and functional brain features throughout the brain aging process.

Recently, fusion techniques for brain age estimation have integrated information from neuroimaging modalities. Traditional methods like concatenation and early fusion may overlook modality specifics, leading to overfitting [35]. Middle fusion, like canonical correlation analysis (CCA), seeks common representations but may miss critical information [36]. Late fusion reduces overfitting but may limit performance by ignoring modality interactions [37]. Advanced deep learning, like autoencoders and variational autoencoders (VAEs), models complex interactions but faces generalizability challenges [38]. Tensor-based fusion captures higher-order relationships but is computationally demanding [39].

To overcome these challenges, our study introduces a low-rank tensor fusion approach. This approach employed low-rank tensors for multimodal fusion, enhancing the accuracy of brain age predictions by integrating structural and functional features. Specifically, we assessed the low-rank tensor fusion technique on both structural and functional brain features, comparing the effects of fused versus non-fused features within our brain age prediction model. Our findings demonstrated that our model performs comparably to state-of-the-art models across three multimodal tasks evaluated on public datasets.

## 2. Materials and Methods

### 2.1. Dataset and Data Availability

We used data from the Cambridge Center for Aging Neuroscience (Cam-CAN) [40,41]. Further details are available at https://camcan-archive.mrc-cbu.cam.ac.uk//dataaccess/; accessed on 15 February 2024. Table 1 summarizes the imaging parameters.

**Figure 1:**
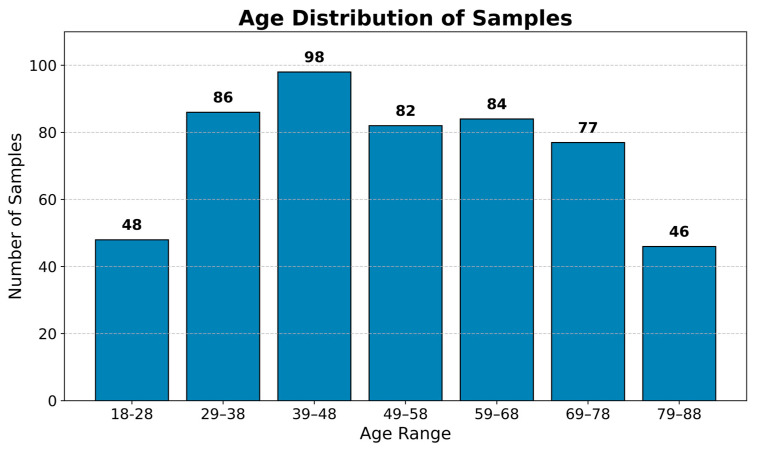
The age distribution of the participants.

In this study, we utilized neuroimaging data from three modalities: sMRI, MEG, and DTI. A total of 521 HCs (270 males, 251 females, aged 18–88, mean age: 52.3 ± 17.7) underwent MR imaging using a 3T scanner. Figure 1 illustrates the age distribution of the participants included in the study. Resting-state MEG data were acquired using a 306-channel system (102 magnetometers, 204 planar gradiometers) with a sampling rate of 1 kHz for 8 min and 40 s with eyes closed. The acquisition parameters were as follows: Flip angle = 9°, field of view = 256 × 240 × 192 mm^3^, voxel size = 1 mm.

### 2.2. Neuroimaging Data Processing

#### 2.2.1. sMRI Data Preprocessing

sMRI images were preprocessed using SPM12 for affine registration, realignment, bias correction, and white matter (WM)/gray matter (GM)/cerebrospinal fluid (CSF) segmentation. CAT12 toolbox (Version 12.9; https://neuro-jena.github.io/cat/index.html accessed on 9 December 2024) was used for estimating WM and GM probability maps with default settings [42]. Skull stripping and registration to standard space were performed using the Montreal Neurological Institute (MNI) 152 template. Following tissue segmentation and bias correction, probability maps of WM, GM, and CSF [43] were generated.

#### 2.2.2. MEG Data Preprocessing

The MEG data were preprocessed using temporal extension (tSSS) in Elekta Neuromag MaxFilter v2.2 for independent head motion correction and noise reduction, with a correlation limit of 0.98 and a 10-s correlation window [44]. Subsequently, Brainstorm [45] was utilized for further MEG data processing, following the procedure described in Niso et al. [46]. High-pass and notch filters were applied at 0.3 Hz and 60 Hz and harmonics, respectively. Cortical surface reconstruction from sMRI was performed using the recon-all algorithm in FreeSurfer (Version 6; https://surfer.nmr.mgh.harvard.edu/ accessed on 9 December 2024) [47,48,49]. After the completion of source reconstruction, the computation of the power spectral density (PSD) was performed encompassing the entire duration of the resting-state scan.

#### 2.2.3. Diffusion MRI Data Preprocessing

The diffusion MRI (dMRI) analyses were conducted using SPM12 with the aa 4.2 pipelines [50] and modules [51]. In the DTI stream, the data underwent skull-stripping using the Brain Extraction Tool (BET) utility in FMRIB’s Software Library (FSL; https://fsl.fmrib.ox.ac.uk/fsl/docs/#/ accessed on 9 December 2024). Later, a parallel branch was employed to nonlinearly estimate the second-order diffusion tensor and its metrics (i.e., fractional anisotropy (FA), mean diffusion (MD), axial diffusion (AD), etc.).

### 2.3. Multimodal Fusion Model

#### 2.3.1. Problem Modeling

**Figure 2:**
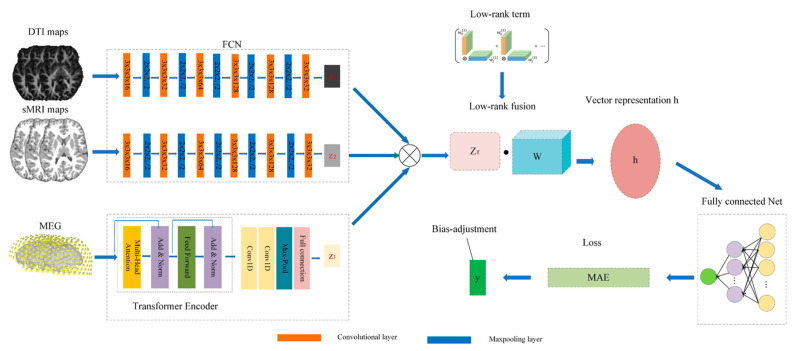
The overview of our proposed approach to fusing multimodal features to predict brain age.

As shown in Figure 2, we first extracted high-level abstract features for multiple modalities. For the extraction of DTI and sMRI features, in order to save computational resources and adapt to neuroimaging datasets with less data, we utilized two identical simple fully convolutional networks (SFCNs) [52] to obtain DTI and sMRI features. Each SFCN comprised six parts. The first five parts contained a 3D convolutional layer with 3 × 3 × 3 convolutional kernels (with channel numbers 32, 64, 128, 256, 256), followed by a batch normalization layer, a 2 × 2 × 2 maximum pooling layer, and was activated using the Rectified Linear Unit (ReLU) function. The sixth part consisted of a 3D convolutional layer with a 1 × 1 × 1 convolutional kernel size and 64 channels, a batch normalization layer, activated using the ReLU function, and finally, a 3 × 4 × 3 average pooling layer. To extract MEG features, we incorporated different attention values for each brain region using the Transformer Encoder [53]. Next, we employed two 1-dimensional convolutional layers with a convolutional kernel size of 1 and channel numbers of 128 and 32, respectively, as well as an average pooling layer to capture the local information from neighboring time points and summarize them [54,55]. Then, a fully connected layer with 64 neurons was used for dimensionality reduction to obtain the extracted MEG features. Finally, a layer with low-rank tensor fusion was added before the fully connected layer. Brain age was estimated from brain images of subjects by feature extraction, low-rank tensor fusion of multimodal features, and mapping with chronological age as label.

#### 2.3.2. Tensor Fusion and Representation

Tensor representation is a successful approach for multimodal fusion. Prior research has indicated that this method outperforms basic concatenation or pooling strategies in capturing multimodal interactions [56,57]. The tensor *Z* is computed by the following:

(1) $$Z={⨂}_{m=1}^{M}z_{m},\;z_{m}\in R^{d_{m}}$$

*M* represents the total number of input modalities and *z_i_* (*i* = 1, 2, 3, …, *M*) represents the features extracted from the multimodal data. The tensor outer product is denoted by ${⨂}_{m=1}^{M}$. The resulting feature after fusion is denoted by *Z_T_*. The tensor *Z* is then fed into a linear layer *g* (⋅), which produces a vector representation as follows:

(2) h= g (Z; W, b)= W·Z+b

where W is the weight of this layer and *b* is the bias. With Z being an order-*M* tensor. In the tensor dot product W·Z, the weight W can be partitioned into $\tilde{W_{k}}$, *k* = 1, …, *d_m_*. Each $\tilde{W_{k}}$ contributes to one dimension in the output vector *h*, i.e., $h_{k}=\tilde{W_{k}}\cdot Z$.

The specific process is as Algorithm 1:
**Algorithm 1.** Multimodal low-rank tensor fusion algorithm.Input: sMRI maps; DTI maps; MEG; label: chronologic age yOutput: brain age $\hat{y}$
Parameters: rank, drop rate η
1: sMRI maps and DTI maps were processed using FCN to extract the spatial structure features $z_{1}\;and\;z_{2}$
2: The PSD extracted using MEG are passed through the Encoder of Transformer to extract brain temporal features $z_{3}$
3: Low-rank fusion $Z_{T}$
4: The fusion feature vector expressed as *h* = *g* (Z; W, *b*) = W·Z + *b*5: Access a fully connected network6: Minimize the loss function *MAE*7: Output bias corrected values for brain age

### 2.4. Model Implementation and Validation

To reduce the reliance on the disparity in brain age (brain age gap = predict brain age − chronological age) on age, a bias correction was applied [58]. To evaluate the model, we used $R^{2}$, root mean square error (*RMSE*), and *MAE* as metrics.

(3) $$MAE=\frac{1}{n}(\sum_{i=1}^{n}\left|y_{i}-Y_{i}\right|)$$

(4) $$RMSE=\sqrt{\frac{1}{n}\sum_{i=1}^{n}{\left(y_{i}-Y_{i}\right)}^{2}}$$

(5) $$R^{2}=1-\frac{\sum_{i=1}^{n}{\left(y_{i}-Y_{i}\right)}^{2}}{\sum_{i=1}^{n}{\left(y_{i}-\bar{y}\right)}^{2}}$$

The model is implemented in Python 3.7 and Pytorch1.11.0 library and was executed on the Ubuntu 18.04 operating system. Throughout the training period, we utilized *MAE* as the loss function with the Adam optimizer [59] using a learning rate of 1 × 10^−4^ and weight decay of 1 × 10^−8^. Additionally, we employed a mini-batch size of 12 and trained for a total of 300 epochs. When the model performs best on the validation set, we save it as the final model and use it for testing.

To evaluate the model, 521 subjects were randomly divided into training (416), validation (52), and testing (53) groups.

## 3. Results

### 3.1. Estimation Based on Different Features and Fusion Methods

The results of different features and fusion methods on the dataset are presented in Table 2. In summary, our multimodal low-rank fusion method generally outperforms the unimodality. Specifically, we designed the low-rank fusion module to combine multimodal features, resulting in a lower *MAE* and higher *R*^2^, while the competing unimodal-based methods achieved an optimal *MAE* of 4.54 and *R*^2^ = 0.92, respectively. Our multimodal low-rank fusion model achieves smaller age errors compared to other non-fusion models.

We employ various combinations of multimodal features to predict brain age. As depicted in Table 2, the prediction model achieves optimal performance when fusing sMRI, FA, and PSD. The subsequent analyses are based on the prediction results from the fusion model that utilized the optimal feature combination. Moreover, we compared traditional feature fusion methods, such as addition or concatenation of these features, and found that our low-rank tensor fusion outperformed these traditional methods in predicting brain age.

### 3.2. Estimation Based on Low-Rank Tensor Fusion Method

**Figure 3:**
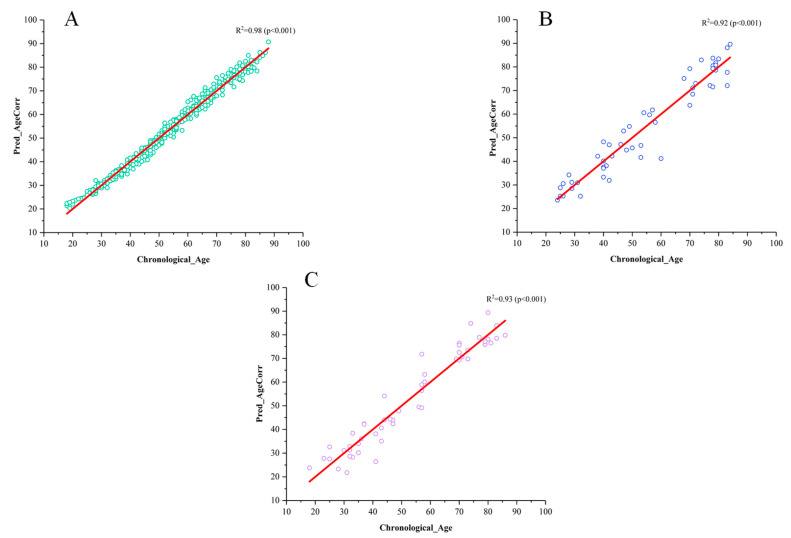
Model performance on each dataset. (**A**) Is the model performance on the training set. (**B**) Is the model performance on the validation set. (**C**) Is the model performance on the test set.

In Table 2, the results demonstrate the effectiveness of our low-rank tensor fusion approach for age prediction. In the training set, we achieved an *R*^2^ value of 0.98, with a *MAE* of 2.25 years and *RMSE* of 2.85 years (refer to Figure 3A). This indicates the successful fusion of features in improving age prediction accuracy. Furthermore, on the validation set, our method yielded an *MAE* of 4.49 years, *R*^2^ of 0.92, and *RMSE* of 5.72 years (refer to Figure 3B). On the test set, we obtained an *MAE* of 4.20 years, *R*^2^ of 0.93, and *RMSE* of 5.43 years (refer to Figure 3C).

## 4. Discussion

Multimodal brain imaging data are extensively utilized for estimating brain age across various contexts. Niu et al. explored different analysis strategies for brain age prediction using large datasets encompassing sMRI, DTI, and fMRI data [60]. Similarly, De Lange et al. utilized machine learning and multimodal imaging data to predict brain age, encompassing gray matter, white matter, and resting-state functional connectivity [61]. Their findings highlighted improved prediction accuracy with the inclusion of multimodal features in the model. Rokicki et al. utilized T1 and T2 structural imaging data, along with cerebral blood flow data from arterial spin labeling, to develop a multimodal model for estimating brain age [62]. Their study demonstrated that integrating multiple types of data can enhance the accuracy of brain age prediction.

We aimed to test whether the use of multimodal neuroimaging data can improve the accuracy of predicting brain age and how to fuse features more effectively. As shown in Table 2, when single-mode features were used to predict brain age, sMRI performed better than either DTI or MEG data (*MAE* = 4.54 years, *RMSE* = 5.52 years, *R*^2^ = 0.92 based on structural data vs. *MAE* = 13.52 years, *RMSE* = 15.68 years, *R*^2^ = 0.57 based on DTI data). When predicting brain age based on MEG data, the depth prediction model did not converge, so the prediction results were not reported. As a result, multimodal data improved prediction performance, as we hypothesized. Specifically, when unimodal data were used to predict brain age, sMRI performed best. This may be because sMRI can more easily capture brain anatomical changes and structural variations in the brain [11], which may better reflect aging [63,64,65,66]. Therefore, the majority of studies used sMRI data to estimate brain age [30,67].

Despite the macroscopic nature of morphological features derived from sMRI data, their sensitivity to neurodevelopmental microstructural changes is limited [68,69]. To enhance model predictive performance, integration of additional modalities is crucial. For example, diffusion MRI techniques, which are adept at capturing tissue microstructure by tracking water molecule diffusion and probing cellular-level environments, offer promising insights [70]. While numerous studies have effectively utilized DTI data to predict brain age, dMRI faces technical challenges and exhibits higher variability compared to conventional modalities like T1- and T2-weighted imaging [71,72]. These intricacies can introduce nonlinear distortions in the original images, affecting diffusion metrics like MD and FA [72], which can reduce the performance of the prediction model. This may also be one of the reasons why the *MAE* is larger when using DTI prediction alone. In the application of functional data, improvements in the prediction of brain age using fMRI are limited by the hysteresis of the hemodynamic response function [73]. However, the MEG with high spatial and temporal resolution can provide complementary features related to normal aging. In exploring MEG data for brain age estimation, we found that the prediction model failed to converge stably, highlighting common challenges in deep learning with complex feature sets, especially during extraction and training. In a previous study, PSD features combined with a machine learning regression model were used to predict brain age, and the *MAE* value was obtained [30]. This is something we need to consider improving in the future.

In this study, we use resting-state MEG (magnetoencephalography), which is preferred over task-based MEG for studying age-related brain changes because it captures the brain’s spontaneous neural activity without the influence of external tasks [74]. This provides a clearer picture of the brain’s intrinsic functional organization and baseline neural efficiency. Unlike task-based paradigms, which can introduce variability due to individual differences in task performance and cognitive strategies, resting-state MEG offers a more stable and reliable measure of brain connectivity, especially in key frequency bands like alpha and beta, which are sensitive to aging.

Compared to fMRI, resting-state MEG has several advantages. First, MEG offers high temporal resolution, capturing brain activity on the millisecond scale, while fMRI operates on a much slower, second-level timescale, potentially missing important rapid oscillatory patterns [75]. MEG also directly measures neuronal activity through magnetic fields, while fMRI relies on the slower BOLD signal, which is influenced by hemodynamic processes rather than direct neural firing [76]. Furthermore, MEG is less sensitive to motion artifacts, providing clearer data, especially for older adults who may have difficulty remaining still. These advantages make MEG particularly well-suited for detecting age-related functional changes in the brain [74].

During the feature extraction phase, we opted for the SFCNs technique to extract features from sMRI and DTI data due to its exceptional ability to capture both local and hierarchical spatial patterns necessary for analyzing brain structure. SFCNs can identify detailed patterns in both gray and white matter across different brain regions, which is crucial for detecting age-related changes. Unlike traditional CNNs, SFCNs preserve spatial resolution throughout the network, meaning they can extract fine-grained features without losing information during down sampling. This ability makes SFCNs particularly well-suited for working with sMRI and DTI data, where maintaining spatial accuracy is important. Research has shown that SFCNs outperform other methods, like traditional CNNs, in extracting structural features from brain imaging data [77].

When FCN and Transformer Encoder were used to process the data and then used to predict brain age after low-rank tensor fusion, the prediction performance of the model was significantly improved. Notably, the fusion of sMRI, FA, and PSD features achieved the highest prediction ability. This is inseparable from the advantages of FCN. Moreover, the Transformer method was introduced by [53], which is mainly based on self-attention and has been applied to many tasks, such as natural language processing, classification tasks [78,79], and brain age prediction [80,81,82,83]. This is because the feature extraction capability of the Transformer method is superior to that of the Recurrent Neural Network, and the source and target sequences can be “self-associated” with each other. In this way, the information contained in the representation of the source and target sequences is richer, and subsequent layers of feed-forward networks improve the representation of the model. These advantages have enhanced the performance of our models.

Our fusion mechanism, which employs low-rank tensor fusion, allows us to utilize tensor rank minimization to learn tensors that more precisely capture the true correlations and underlying structures within multimodal data, effectively reducing input errors [84,85]. Studies have shown that FA is the most age-sensitive of the conventional DTI metrics [86]. This may be one of the reasons why our prediction model with FA performs better in feature fusion. In contrast to the conventional approach of fusing multiple modes of features, the *MAE* value is reduced, and the prediction result is more desirable. Table 3 summarizes the current studies using Cam-CAN data to predict brain age as well as our proposed method. Specifically, Xifra-Porxas, Alba, et al. [30] used dimensionality reduction techniques and Gaussian process regression (GPR) to predict brain age. Using MEG features (*MAE* = 9.60 years) produced worse performance than using MRI features (MA =5.33 years), but a stacked model combining the two features improved age prediction performance (*MAE* = 4.88 years). Popescu, Sebastian G et al. [87] have trained a U-Net model that utilizes deep learning techniques to generate individualized 3D brain maps at a local level for age prediction, which could provide spatial information about anatomical patterns of brain aging. The Cam-CAN data were then tested on the model and the *MAE* was 9.5 years. Han, Juhyuk et al. [88] trained and compared the predictive performance of 27 machine learning models for brain age prediction and applied the trained models to the Cam-CAN dataset. The *MAE* and *R*^2^ were 7.08–10.50 years and 0.64–0.85, respectively. A brain age prediction model was constructed by using the transfer learning method and a large dMRI dataset as the source domain. Then, the trained model was used to test Cam-CAN data, and the *MAE* was 4.68–5.71 years [89]. From Table 3, we can see that our proposed method has achieved a better performance than that of other previous studies.

Recently, with the broad application of multimodal data in brain age prediction, numerous advanced multimodal fusion methods have been proposed and have achieved promising results. For instance, Clements RG et al. leveraged a multimodal 3D convolutional neural network and magnetic resonance elastography (MRE) technology to predict brain age. The advantages of their method lie in the innovative combination of these two technologies, achieving high-precision brain age prediction, and further enhancing prediction accuracy through multimodal fusion, offering possibilities for the early diagnosis of neurodegenerative diseases. However, this method also has some drawbacks, including high model complexity, substantial computational resource requirements, strong data dependency, and limitations such as no performance improvement when incorporating damping ratio into the model [90]. The multimodal Transformer-based architecture proposed by Wang J and his team demonstrates notable advantages in biological age prediction, including improved prediction accuracy through the fusion of facial, tongue, and retinal images, as well as its potential application in risk stratification and progression prediction of chronic diseases. However, this method also faces some challenges, such as the heterogeneity of the aging process limiting prediction accuracy, deployment difficulties due to technical complexity, and considerations regarding personal privacy and ethical issues [91].

Compared with these methods, our proposed low-rank tensor fusion approach demonstrates notable advantages in multimodal brain age prediction tasks. First, our method leverages low-rank tensor decomposition to effectively reduce redundant information within multimodal data, thus enhancing computational efficiency. Second, due to the automatic selection of salient features between modalities afforded by low-rank tensor decomposition, our method exhibits greater robustness under data imbalances [92]. Additionally, in terms of cross-dataset generalization, the low-rank tensor fusion method adapts better to feature differences across datasets, demonstrating high adaptability [93]. Additionally, by utilizing the low-rank tensor fusion technique, the likelihood of overfitting is minimized, while interpretability is enhanced through the extraction of crucial shared features instead of learning noise specific to each modality. This method has proven to be successful in various multimodal learning tasks, including the classification of neurodegenerative diseases [39], underscoring its resilience and efficacy.

In terms of potential clinical applications, our multimodal neuroimaging approach for brain age prediction holds promise in identifying individuals at risk of neurodegenerative diseases or monitoring disease progression. For instance, deviations between predicted and chronological brain age, known as brain age gaps, have been shown to serve as biomarkers for various neurological conditions, including dementia and other conditions [4,63]. By leveraging the improved prediction accuracy achieved through multimodal data integration, our model could potentially offer earlier and more accurate insights into brain health, facilitating timely interventions and personalized treatment strategies. We plan to explore these clinical implications in future studies.

Our study has several limitations. First, the sample size and the lack of an independent dataset for assessing the generalizability of our model are notable constraints. Our primary aim was to test the low-rank tensor fusion algorithm tailored for deep learning-based frameworks in brain age estimation. We used the Cambridge Center for Aging Neuroscience (Cam-CAN) dataset, which includes sMRI, DTI, and resting-state MEG data. While validating our results on an independent dataset could strengthen the findings, it is challenging to find a dataset that includes all three modalities, especially resting-state MEG data. Therefore, future studies should validate the proposed algorithm on larger, independent datasets. Another limitation involves the failure of our deep learning model to converge during training when using MEG data. This may have been due to issues such as improper weight initialization, inappropriate learning rates, insufficient data, overfitting, or a non-convex loss function. Despite adjusting factors like learning rate and weight initialization, the model did not converge using MEG data alone. However, the model did show convergence when combining MEG features with DTI and sMRI data. Future research with larger sample sizes is needed to investigate these convergence issues and propose solutions for deep learning models applied to brain imaging data. Additionally, in this study, we compared our low-rank tensor fusion algorithm with single-input data and traditional feature fusion methods, such as addition or concatenation. Our results demonstrated that the low-rank tensor fusion method provided better prediction accuracy than traditional feature fusion methods. Future studies could focus on developing more accurate deep learning-based data fusion methods and comparing them to existing techniques. It is important to note that the choice of data fusion method in deep learning models depends on factors such as data characteristics (e.g., structured, unstructured, multimodal), model complexity, and computational resources [94,95]. Finally, we intended to assess our low-rank fusion method using combinations of all feature maps but were limited by computational resources. Future studies may be able to explore this approach further.

## 5. Conclusions

In this study, we presented a novel low-rank tensor fusion algorithm developed to integrate multimodal brain imaging data for the purpose of brain age estimation. Our strategy involves integrating three different imaging techniques—sMRI, DTI, and resting-state MEG—in order to offer a more thorough understanding of brain aging. We evaluated the method using the Cambridge Centre for Aging Neuroscience (Cam-CAN) dataset. The results indicated that incorporating both structural and functional brain features enables our model to offer a deeper understanding of the brain’s aging process. Our data fusion method exhibited performance that rivals state-of-the-art techniques in different multimodal tasks, as tested on datasets that are publicly accessible.

## Figures and Tables

**Table 1:** The imaging parameters of different neuroimaging data used for brain age modeling.

Scans Type	Sequence	TR (ms)	TE (ms)	Flip Angle (°)	FOV (mm)	Voxel Size (mm)
sMRI	MPRAGE	2250	2.99	9	256 × 240 × 192	1 × 1 × 1
Diffusion-weighted		9100	104		192 × 192	2 × 2 × 2
	Sampling rate (HZ)		Duration (min:s)		Task	
Resting-state MEG	1000		08:40		Rest with eyes closed	

Abbreviations: sMRI, structural magnetic resonance imaging. TR, the Alzheimer’s Disease Neuroimaging Initiative. TE, Echo Time. MPRAGE, Magnetization Prepared-Rapid Gradient Echo imaging.

**Table 2:** Performance metrics for various features and fusion methods.

	Strategies	Feature	*MAE* (y)	*RMSE* (y)	*R* ^2^	*p*
Training set	Unimodal	DTI	11.67	12.13	0.74	<0.001
		MEG	-	-	-	-
		sMRI	3.83	4.72	0.94	<0.001
	Traditional fusion	Add	2.89	4.30	0.96	<0.001
		Concat	3.11	4.43	0.96	<0.001
	Low-rank tensor fusion	sMRI + AD + PSD	2.30	2.94	0.96	<0.001
		sMRI + MD + PSD	3.04	3.82	0.95	<0.001
		**sMRI + FA + PSD**	**2.25**	**2.85**	**0.98**	<0.001
Validation set	Unimodal	DTI	12.69	14.56	0.71	<0.001
		MEG	-	-	-	-
		sMRI	4.91	5.61	0.91	<0.001
	Traditional fusion	Add	7.91	9.29	0.89	<0.001
		Concat	10.65	12.17	0.79	<0.001
	Low-rank tensor fusion	sMRI + AD + PSD	5.32	6.67	0.90	<0.001
		sMRI + MD + PSD	4.80	6.18	0.90	<0.001
		**sMRI + FA + PSD**	**4.49**	**5.72**	**0.92**	<0.001
Testing set	Unimodal	DTI	13.52	15.68	0.57	<0.001
		MEG	-	-	-	-
		sMRI	4.54	5.52	0.92	<0.001
	Traditional fusion	Add	8.56	10.13	0.90	<0.001
		Concat	11.36	13.46	0.72	<0.001
	Low-rank tensor fusion	sMRI + AD + PSD	4.59	5.90	0.91	<0.001
		sMRI + MD + PSD	4.47	5.45	0.92	<0.001
		**sMRI + FA + PSD**	**4.20**	**5.43**	**0.93**	<0.001

Abbreviations: *MAE*, mean absolute error. *RMSE*, root mean square error. *R*^2^, the coefficient of determination. DTI, diffusion tensor imaging. MEG, magnetoencephalography. sMRI, structural magnetic resonance imaging. AD, axial diffusivity. MD, mean diffusivity. FA, fractional anisotropy. PSD, power spectral density. The symbol “-” indicates that this result is not reported. Add: a parallel strategy to combine the two feature vectors into a compound vector. Concat: a series of feature fusion methods, directly linking the features. The prediction results using MEG data are not reported because the depth prediction model did not converge.

**Table 3:** Comparative results of brain age estimation on Cam-CAN data.

Studies	Modal	*MAE* (y)	*R* ^2^
[30]	sMRI, MEG	4.88–9.6	-
[87]	sMRI	9	-
[88]	sMRI	7.08–10.50	0.64–0.85
[89]	dMRI	4.68–5.71	-
**Our method**	sMRI, MEG, DTI	**4.20**	**0.93**

Abbreviations: *MAE*, mean absolute error. *R*^2^, the coefficient of determination. DTI, diffusion tensor imaging. MEG, magnetoencephalography. sMRI, structural magnetic resonance imaging. dMRI, diffusion magnetic resonance imaging.

## Data Availability

We used data from the Cambridge Center for Aging Neuroscience (Cam-CAN): https://camcan-archive.mrc-cbu.cam.ac.uk//dataaccess/ (15 February 2024).
